# Assessing the Comparability of Paper and Electronic Versions of the EORTC QOL Module for Head and Neck Cancer: A Qualitative Study

**DOI:** 10.2196/cancer.7202

**Published:** 2017-05-12

**Authors:** Josephine Norquist, Diana Chirovsky, Teja Munshi, Chloe Tolley, Charlotte Panter, Adam Gater

**Affiliations:** ^1^ Merck & Co., Inc. North Wales, NJ United States; ^2^ Merck & Co., Inc. Kenilworth, NJ United States; ^3^ Adelphi Values Ltd Patient-Centered Outcomes Bollington, Cheshire United Kingdom

**Keywords:** ePRO, comparability, EORTC QLQ-C30, head and neck cancer, quality of life questionnaire

## Abstract

**Background:**

Patient-reported outcome (PRO) instruments are important tools for monitoring disease activity and response to treatment in clinical trials and clinical practice. In recent years, there have been movements away from traditional pen-and-paper PROs towards electronic administration. When using electronic PROs (ePROs), evidence that respondents complete ePROs in a similar way to their paper counterparts provides assurance that the two modes of administration are comparable or equivalent. The European Organisation for Research and Treatment of Cancer (EORTC) Quality of Life Questionnaire-Core 30 item (EORTC QLQ-C30) and associated disease-specific modules are among the most widely used PROs in oncology. Although studies have evaluated the comparability and equivalence of electronic and original paper versions of the EORTC QLQ-C30, no such studies have been conducted to date for the head and neck cancer specific module (EORTC QLQ-H&N35).

**Objective:**

This study aimed to qualitatively assess the comparability of paper and electronic versions of the EORTC QLQ-H&N35.

**Methods:**

Ten head and neck cancer patients in the United States underwent structured cognitive debriefing and usability interviews. An open randomized crossover design was used in which participants completed the two modes of administration allocated in a randomized order. Using a “think-aloud” process, participants were asked to speak their thoughts aloud while completing the EORTC QLQ-H&N35. They were thoroughly debriefed on their responses to determine consistency in interpretation and cognitive process when completing the instrument in both paper and electronic format.

**Results:**

Participants reported that the EORTC QLQ-H&N35 demonstrated excellent qualitative comparability between modes of administration. The proportion of noncomparable responses (ie, where the thought process used by participants for selecting responses appeared to be different) observed in the study was low (11/350 response pairs [35 items x 10 participants]; 3.1%). Evidence of noncomparability was observed for 9 of the 35 items of the EORTC QLQ-H&N35 and in no more than 2 participants per item. In addition, there were no apparent differences in level of comparability between individual participants or between modes of administration.

**Conclusions:**

Mode of administration does not affect participants’ response to, or interpretation of, items in the EORTC QLQ-H&N35. The findings from this study add to the existing evidence supporting the use of electronic versions of the EORTC instruments when migrated to electronic platforms according to best practice guidelines.

## Introduction

Patient-reported outcomes (PROs) are used in clinical trials and clinical practice to assess symptoms, impacts, and health-related quality of life (HRQoL) from the patient perspective. Understanding patients’ symptoms and physical functioning is important in oncology and other disease areas to assess disease activity and response to treatment, and this information best comes from the patients themselves [1]. The European Organisation for Research and Treatment of Cancer Quality of Life-Core 30 (EORTC QLQ-C30) is a self-reported 30-item questionnaire developed to assess HRQoL in cancer participants [2]. It was established in 1987 and has been used in over 3000 studies worldwide [3].

Tumor-specific questionnaire modules supplement the EORTC QLQ-C30, including the EORTC Quality of Life Head and Neck 35-item questionnaire (EORTC QLQ-H&N35). Head and neck cancer is the sixth most common cancer worldwide, with an incidence of over 600,000 newly diagnosed cases each year [4]. The disease and associated treatments can have a profound effect on patients’ HRQoL [5]. Both the EORTC QLQ-C30 and EORTC QLQ-H&N35 were originally developed and validated for administration and completion via pen and paper. However, there are considerable advantages to the adaptation of PRO measures to electronic forms of data capture. This includes the potential for minimizing administrative burden, thereby increasing patient acceptance and adherence, avoiding secondary data entry errors, and ultimately producing more accurate and complete data [6-11]. However, in migrating pen-and-paper instruments to electronic platforms, some adaptations and modifications are necessary. Evidence that respondents complete electronic PROs (ePROs) in a similar way to their paper counterparts and that the two modes of administration may be considered comparable or equivalent is desirable and is a requirement if the electronic instrument is to be used to support regulatory labeling claims [12,13]. The concern with implementing an electronic mode of administration for a previously developed and validated instrument for paper-and-pen completion within a clinical trial is that measurement error could be introduced if the electronic version of the instrument PRO does not provide data comparable to the original paper version. This would reduce statistical power and interfere with the ability of the trial to detect real change (ie, treatment effect) in the PRO-based endpoint [12,14].

A number of meta-analyses and systematic reviews of studies evaluating measurement equivalence between ePROs and their validated paper-based equivalents in a number of disease areas have been conducted [15-17]. Findings are supportive of the comparability between paper and electronic modes of administration [15-17], and studies have reported a general preference among respondents for electronic administration [15]. Prior studies have evaluated the comparability of paper and electronic versions of the EORTC QLQ-C30 and have shown good levels of comparability [9,11,17,18]. However, no studies evaluating the comparability of paper and electronic versions of the EORTC QLQ-H&N35 (as a companion module to the EORTC QLQ-C30, for use with head and neck cancer participants) have been published.

This study aimed to provide evidence on the qualitative comparability of data collected from paper versus electronic (tablet-based) administration of the EORTC QLQ-H&N35. The primary objective was to explore whether there were any features of the electronic-version of the EORTC QLQ-H&N35 where participants’ understanding and interpretation of instructions, items, and response options differed when compared to the original pen-and-paper version of the instrument. While the primary objective relates to the EORTC QLQ-H&N35, participants also completed the EORTC QLQ-C30 in line with developer guidelines. Feedback from participants regarding the usability of electronic device for completion of the instruments was also investigated.

## Methods

The level of evidence required to assess comparability across modes of administration depends on the extent to which the instrument has been modified from its original format in migration to the new format [12,13]. Moving from a pen-and-paper format to an electronic screen text format without significantly reducing font size, altering item content, recall period, or response options (including appreciation of the fact that ePRO versions may present fewer items on a screen than are typically presented on a page) may be considered a “minor modification” [12]. Evidence suggests that such modifications are unlikely to have a substantive effect on the performance of the measure. Nonetheless, evidence that respondents interpret instruments in the same manner as the original paper version and that electronic administration is suitable for the intended population is recommended by best practice guidelines [12]. Based on the minor nature of the changes to electronic format, comparability can be assessed through qualitative research methods (cognitive debriefing interviews and usability testing) with the focus on comparability of the “thought processes” used to respond to items, rather than a quantitative assessment of equivalence of instrument scores.

**Figure 1:**
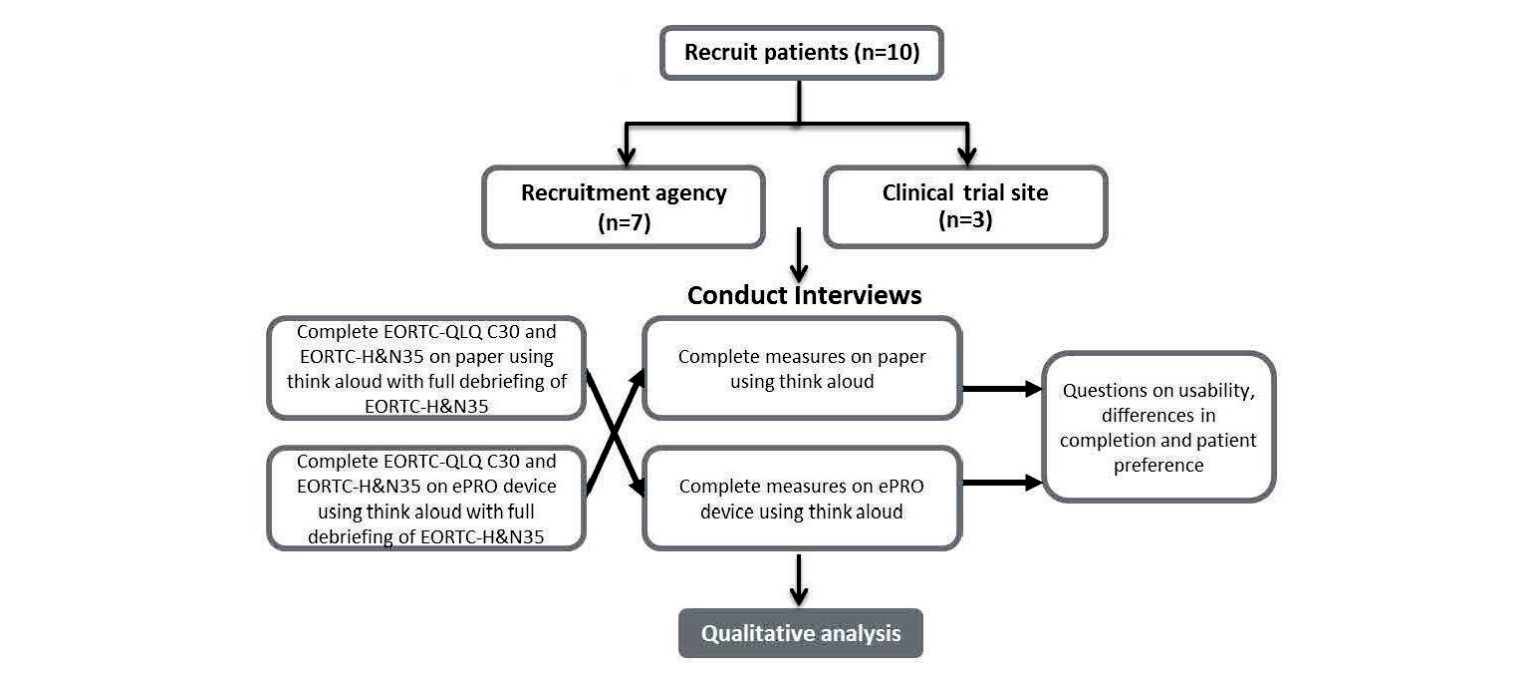
Interview methodology.

The EORTC QLQ-H&N35 is designed to be administered alongside the EORTC QLQ-C30. This study implemented an open randomized crossover design in which participants completed the two modes of administration for the EORTC QLQ-C30 and H&N35 (Group 1 [G1]: paper followed by electronic tablet; Group 2 [G2]: electronic tablet followed by paper) allocated in a randomized order (Figure 1). The design allowed the researchers to ensure the order of administration had no influence on the results. Participants completed each mode of administration one after another (ie, no break between completions). The interview process itself acted as a distraction task by incorporating interviewer questioning on each item as the participant completed the instruments to minimize potential learning effects.

### Ethics

This study was approved by Copernicus Group, a centralized Institutional Review Board in the United States (IRB #ADE1-15-702). The study was performed in accordance with the ethical standards in the 1964 Declaration of Helsinki and its later amendments.

### Recruitment

Ten people with head and neck cancer were recruited to participate into this study in the United States. This sample size is in accordance with recommendations from the International Society for Pharmacoeconomics and Outcomes Research ePRO Good Research Practices Task Force Report, which recommends 5-10 participants for studies involving cognitive debriefing and usability testing where minor modifications have been made in migration to another mode of administration [12].

Participants were required to meet predefined inclusion and exclusion criteria (Table 1). Participants were recruited via a specialist oncology center and a patient advocacy group between May and September 2016. A demographically and clinically diverse sample of participants with head and neck cancer were recruited by monitoring predefined quotas for gender, age, ethnicity, highest education level, technical familiarity, disease severity, and Eastern Cooperative Oncology Group (ECOG) status. These quotas correspond to sample characteristics of previous EORTC QLQ-H&N35 validation studies [19-21] and were used to ensure that the recruited sample was representative of the broader target population.

**Table 1:** Study inclusion/exclusion criteria.

Study	Criteria
Inclusion	Participant is at ≥18 years of age.
	Participant is willing and able to provide written informed consent and attend and participate in a 1-hour interview.
	Participant is literate in English and verbally fluent in English.
	Participant has confirmed H&N cancer or has been in remission for up to 3 years^a^.
	The eligible primary tumor locations included pharynx (oropharynx, hypopharynx, epipharynx, parapharynx, nasopharynx), oral cavity, and larynx.^b^
	Participant has a current ECOG performance status of grade 0-2.^b^
	
	
Exclusion	Participant has brain metastases or intracranial extension of the tumor with cognitive impairment.
	Participant has significant difficulty hearing, reading, or speaking.
	Participant has an uncontrolled psychiatric condition or mental condition (eg, schizophrenia, bipolar disorder) or severe physical, neurological, or cognitive deficits rendering the participant unable to understand study scope or participate in a 1-hour interview.

^a^Consistent with disease criterion defined in validation studies of the H&N module [19].

^b^Participants recruited via the patient advocacy group were asked to provide their tumor location using a descriptive diagram, and ECOG status was estimated based on participant self-report.

### ePRO Device

The electronic tablets used in this study (TrialMax Slate) were provided by a third party agency, CRF Health. The device used was an ACER ASPIRE SWITCH 10, with a 10.1-inch display. As the devices used were “dummy devices,” participant responses were not recorded on the devices themselves but participants were asked to read aloud their responses, with responses recorded by the interviewer and documented on the audio-recording of the interview. The device displayed approximately 6 items per screen. Of note, this format differs from the paper versions of the EORTC QLQ-H&N35, which displays approximately 18 items per page.

### Interview Procedure

All interviews were conducted by trained experienced interviewers using a semi-structured interview guide. In the absence of existing published evidence regarding the comparability of paper and electronic versions, the EORTC QLQ-H&N35 was the primary focus of discussions, although participants also provided feedback on the interpretation of individual items of the EORTC QLQ-C30.

Participants completed the questionnaires on the first mode of administration as part of a “think-aloud” exercise, whereby they were asked to speak aloud their thoughts as they read each instruction and complete each item. Interviewers used focused probes during this process to ensure that the EORTC QLQ-H&N35 was debriefed in full and that participant understanding/interpretation of items and reasons for selecting certain responses was fully understood. Participants then repeated the think-aloud exercise for the second mode of administration. Any apparent differences in interpretation of instrument instructions, items, and response options between modes of administration were explored.

Finally, participants were asked for their feedback on the usability of the electronic tablet, any perceived differences in their experience of completing the instruments across modalities, and their preference for either modality.

### Analysis

All interviews were audio-taped and transcribed verbatim to allow for qualitative analysis using ATLAS.ti software. All transcripts were assigned a unique patient identification code, which was made up of the interview location number, participant number, participant gender, participant age, and group number (ie, G1 being paper followed by electronic version, and G2 being electronic followed by paper version). In accordance with the principles of thematic analysis, a coding scheme was developed in which excerpts from transcripts were assigned codes and grouped according to consistent themes. Coding was completed by one researcher.

A primary focus of the analysis was to determine the extent to which participant responses to PRO items in electronic and pen-and-paper formats could be considered comparable. In this context, responses were defined as comparable if it was clear from qualitative feedback that the participant had interpreted the item and selected their response using the same thought process for both modes of administration. Crucially, while in many cases participants may have selected identical responses to items for each respective administration, this was not necessary for the formats to be considered comparable. Similarly, even if respondents had selected identical responses, if it was clear from discussion and feedback that the thought process for selecting responses was different then this was highlighted as noncomparable. Where it was not clear whether the participant had interpreted the item differently between modalities, this was counted as not clear. Comparability of response was evaluated for each participant by 2 independent researchers and was checked by the project leader.

## Results

### Demographic and Clinical Characteristics

Table 2 contains the demographic and clinical characteristics of the participants who participated in this study. The majority of participants were under 65 (n=9), and an equal number of male (n=5) and female (n=5) participants were recruited. Participants had been diagnosed with head and neck cancer for various lengths of time ranging from less than 6 months (n=3) to more than 2 years (n=4). All disease stages were represented in the sample. The majority of participants (n=8) reported using a touchscreen device “all the time.” Only 2 participants reported not frequently using such type of devices: “sometimes” (n=1) or “rarely” (n=1).

**Table 2:** Demographic and clinical characteristics of participants.

	
Characteristic		Participants, n
**Age, mean (range)**		51.5 (34-80)
	<65 years	9
	>65 years	1
**Gender**		
	Male	5
	Female	5
**Living status**		
	Live alone	2
	Live with husband/wife/partner	4
	Live with parents/family or friends	4
**Ethnicity**		
	Hispanic or Latino	1
	Not Hispanic or Latino	9
**Race**		
	Caucasian	7
	Multiracial	3
**Highest education level**		
	High school diploma	4
	Some years of college	1
	University/college degree (2 or 4 year)	4
	Graduate or professional degree	1
**Work status**		
	Working full or part-time	6
	Retired	2
	Not working due to head and neck cancer	2
**Devices used on a regular basis**		
	Desktop	9
	Tablet	7
	Mobile phone	8
**Touchscreen device use**		
	All the time	8
	Sometimes	1
	Rarely	1
**Time since diagnosis**		
	<6 months	3
	6-12 months	1
	1-2 years	2
	2-3 years	2
	3-5 years	2
		
		
		
		
**Location of primary tumor**		
	Oropharynx	2
	Hypopharynx	1
	Epipharynx	1
	Larynx	3
	Oral cavity	3
**Current disease stage**		
	Stage I	4
	Stage II	2
	Stage III	1
	Stage IVa-b	1
	Stage IVc	2
**Recurrent H&N cancer**		
	Yes	2
	No	8
**In remission?**		
	Yes	3
	No	7
**ECOG performance status**		
	0	6
	1	1
	2	3
**Current active treatment**		
	Concurrent systemic therapy plus radiation	1
	Radiation	1
	Immunotherapy	1
	None	6
	Other^a^	1

^a^Participant due to start a trial in 2 weeks.

### Qualitative Comparability

**Figure 2:**
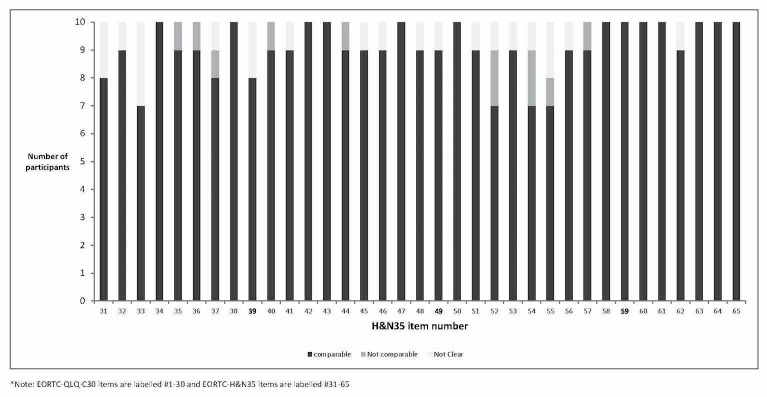
Qualitative comparability of EORTC QLQ-H&N35 (by item).

As the EORTC QLQ-H&N35 is designed to be administered following the EORTC QLQ-C30, numbering of EORTC QLQ-H&N35 items starts at 31. Overall, the EORTC QLQ-H&N35 demonstrated strong evidence of qualitative comparability between modes of administration. The majority of items showed comparability when completed on paper and electronically, with feedback from participants indicating that they had interpreted the item in the same way or used the same thought process when completing the item in both modalities (Figure 2). For example:

(01-03-F-50-G1): Item 31. Have you had pain in your mouth?

Have you had pain in your mouth? I’m going to say a little only because I have dentures and I think, um, that all has a little bit to do with the, the radiation and stuff. I had a hard time wearing them... I’m going to say I had a little bit of discomfort.

Paper

Um, have you had pain in your mouth? I’m going to put a little. Again, that’s just the denture thing.

ePRO

(03-03-F-48-G2): Item 51. Have you had trouble eating in front of other people?

A little bit, yes. I feel a little self-conscious sometimes… I was a little more self-conscious about eating in front of others out in public… because of the partial paralysis I have at the corner of my mouth and it just sort of makes me feel a little awkward.

ePRO

I would choose number two, a little bit. More of a self-conscious thing, uh, than it was mechanical.

Paper

In total, the proportion of noncomparable responses (ie, where the thought process used by participants for selecting responses appeared to be different) observed in the study was low (11/350 response pairs [35 items x 10 participants]; 3.1%). Evidence of noncomparability was observed for only 9 of the 35 items of the EORTC QLQ-H&N35. For these 9 items, noncomparable responses were typically observed only for a single participant (7/9 items) and never more than 2 individual participants (2/9 items). In instances where responses were noncomparable, participants seemed to use a different thought process to select a response (eg, responding to the question in a different context), although participants often selected the same or adjacent response options. Indeed, no instances where response options selected by participants differed by more than one response category between modes of administration were observed (examples provided below). For items 52 (“Have you had trouble enjoying your meals?”) and 54 (“Have you had trouble talking on the telephone?”), where 2 individual participants provided noncomparable responses, the different thought processes used may be attributed to participants’ understanding of item wording:

(03-03-F-48-G2): Item 40. Have you had problems opening your mouth wide?

Have you had problems opening your mouth wide? Uh, quite a bit, number three. Um, biting into sandwiches and, you know, taking spoonfuls of food has been problematic this week.

Paper

Have you had problems opening your mouth wide? I’m going to go with four, uh, very much ‘cause I’m always having problems opening my mouth wide… I think I kind of consider that similar to the have you had pain in your jaw. And, um, those two kind of are related. Uh, opening my jaw wide, opening my mouth wide is painful.

ePRO

(03-02-F-39-G1): Item 52. Have you had trouble enjoying your meals?

Have you had trouble enjoying your meals? I just say a little, only because of the swallowing being little inconvenient, but other than that I really don’t have any problems.

Paper

Have you had trouble enjoying your meals? No, not at all. I enjoy my meals.

ePRO

(01-01-M-59-G1): Item 55. Have you had trouble having social contact with your family?

Have you had trouble having social contact with your family? Not at all. We get together for all the family functions, birthdays, and Christmas and Easter and all that good stuff.

ePRO

Have I had trouble having social contact with my family? I don’t know if this pertains to your survey, but because I am living with my mother right now she is really hard of hearing. And between my voice and her ears I don’t talk very much... I’m going to put a little.

Paper

There were a small number of instances across 17 items where it was not possible to determine whether participant interpretation was comparable (ie, detailed as “not clear”). In the majority of cases, this was due to the brevity of information provided by participants during the think-aloud and subsequent discussion. For example:

(03-02-F-39-G1): Item 33. Have you had soreness in your mouth?

Have you had soreness in your mouth? Uh, number two, a little… just an achy type pain, and I do have that achiness from time to time.

Paper

Have you had soreness in your mouth? One, not at all.

ePRO

**Figure 3:**
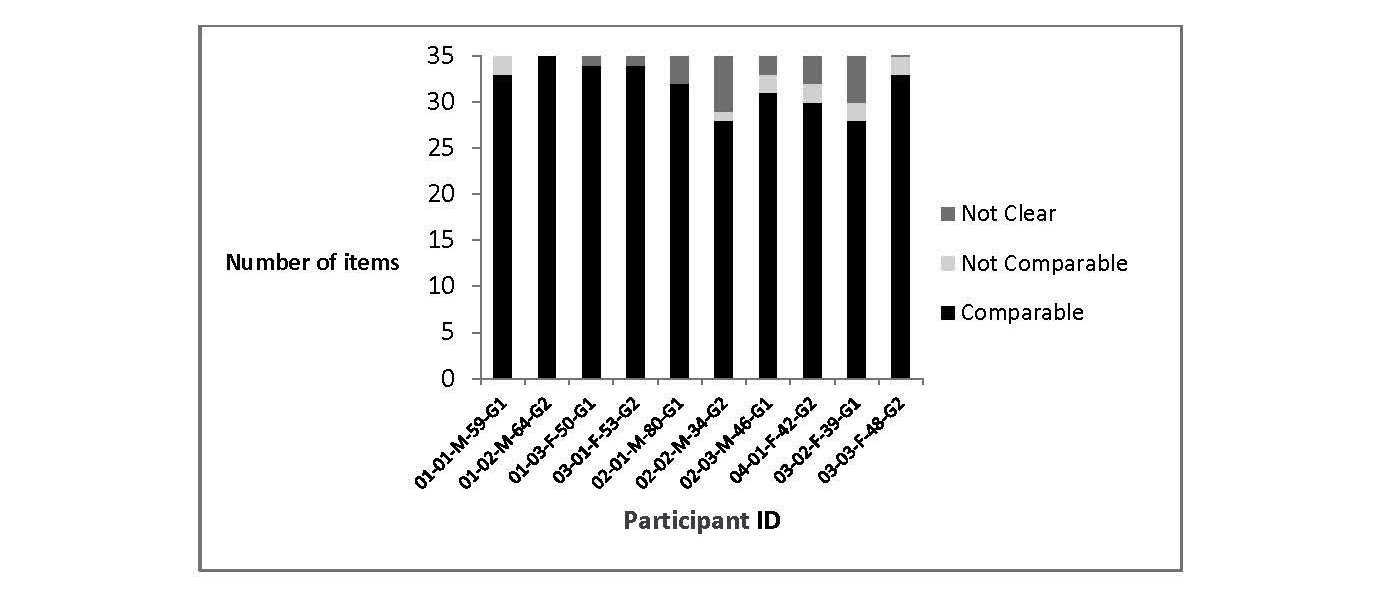
Qualitative comparability of EORTC QLQ-H&N35 (by participant).

Exploring noncomparable responses in more detail revealed that that these came from 6/10 participants. Among these participants, no individual appeared to interpret items differently or use a different thought process to select a response on any more than two items (Figure 3). Discernable differences between those participants demonstrating some evidence of noncomparable responses and the remainder of the study sample, in terms of demographic and clinical characteristics, were not evident. Furthermore, there were no trends to indicate that order of administration (eg, paper followed by electronic and vice versa) had an impact on comparability and there did not appear to be any systematic bias. Of responses to the 35 items on each mode of administration (175 completion pairs for each group), participants completing on paper and then ePRO had 158 instances of equivalence (90%) while participants completing on ePRO and then paper had 160 instances of equivalence (91%).

When asked directly, most participants (n=8) reported that mode of administration (paper or electronic) made no difference in their understanding of the EORTC QLQ-H&N35 instructions and items or the way in which they selected responses to items. Two participants reported that the mode of administration did influence their ability to understand, interpret, and respond to EORTC QLQ-H&N35 items. One participant commented that the tablet version of the instrument was easier to understand: “where in the paper I may have gone through it quicker.” Another participant reported that it “feels differently looking at it on paper.” While no further information was provided by these participants, their responses were largely comparable across modes of administration (equivalent responses provided across 28/35 items, 80%; and equivalent responses provided across 33/35 items, 94%; respectively). While not the purpose of the current study, equivalence of scores on the EORTC QLQ-C30 was also observed.

### Usability

All 10 participants reported that the tablet device was easy to use, with 7 participants spontaneously adding that it was easier than the paper version. No participants demonstrated any issues with selecting a response on the touchscreen or moving on to the next page of the questionnaire. Similarly, participants did not report any concerns about changing an answer, moving to a previous page, or saving responses. Some participants commented that completing the instrument on the electronic tablet was faster than on paper (n=3) or that there was no difference in completion time between paper and electronic tablet version (n=3).

When asked, most participants (n=7) said that they preferred completing the instrument on the electronic tablet than on pen and paper, as it was easier to use (n=3), had a better “flow” (n=2), would be more efficient in data transfer (n=2), and meant that pen and paper were not needed (n=1). Two participants preferred pen and paper, as it was familiar (n=1) and allowed the full questionnaire to be viewed at once (n=1). One participant did not have a preference.

## Discussion

This study used a standard qualitative methodology to assess comparability for the EORTC QLQ-H&N35, whereby comparability was judged to have been met if the participant demonstrated that they had interpreted the item in the same way for both completions. There were only a very small number of instances (3.1%) where participants interpreted the item differently on paper and electronic tablet and used a different thought process to choose a response. Overall, these findings suggest that mode of administration does not affect the way that participants respond to and interpret items in the EORTC QLQ-H&N35. While not the main focus of the study, observations and agreement between scores suggest that paper and electronic versions of the EORTC QLQ-C30 were also comparable. These findings are in alignment with existing literature regarding the EORTC QLQ-C30 and other associated modules [9,11,17,18,22,23]. The methods used to assess comparability in this study are in line with industry-recognized, best practice recommendations on generating evidence for comparability or equivalence when minor changes have been made in migration of an instrument from paper to electronic format [12]. Specifically, where minor modifications are made, industry-accepted best practice standards recommend that small-scale (5-10 patients) cognitive debriefing and usability testing be conducted to establish that participants are responding to the items in the intended manner and that the ePRO software works sufficiently when used by the target population [12]. The qualitative methodology allowed for greater insight into the potential impact of mode of administration on participants’ responses to individual items than would have been obtained from a quantitative study looking at score equivalence.

It is worth noting that there were some limitations to the study design. While there were a small number of instances where participants provided a different response across modes of administration, we acknowledge that this could easily be attributed to participant understanding of item wording or fallibility of human memory rather than the impact of the mode of administration. Furthermore, the interview procedure allowed participants a second opportunity to consider their response and question their original choice. Some participants were aware that they were changing their response between modes of administration. There were some participants who saw the double completion as a memory test and aimed to try and remember their original response on the second mode of administration, rather than treating it as a new item. Finally, it was difficult to recruit participants who were not familiar with using electronic smart devices. This is reflective of the widespread use of smart devices, across all age groups, in the United States in 2016 [24].

Given the large amount of evidence for comparability of electronic and paper versions of the EORTC instruments and the lack of concerns identified, further comparability studies for EORTC modules that have undergone minor modifications to electronic administration are unlikely to lead to different conclusions and are probably not warranted. It is acknowledged that this study explored equivalence of paper versus PROs administered in an electronic (tablet) format, yet there exist other electronic formats (eg, mobile phone or app-based versions) that are commonly implemented. However, meta-analyses and systematic reviews by Gwaltney et al and Muehlhausen et al conclude that the majority of comparability and equivalency studies demonstrate that the paper PRO questionnaires evaluated are quantitatively comparable with measures administered on a variety of electronic devices, including tablets and mobile phones, when minor modifications have been made [16,17]. These findings suggest that electronic measures can generally be assumed to be comparable to pen-and-paper measures and the authors question whether equivalence studies are necessary when an instrument has been migrated to an electronic platform following best practice guidelines for minor modifications [14,16].

### Conclusion

This study provides evidence for comparability of the EORTC QLQ-H&N35 administered via an electronic device compared to administration via pen and paper. These findings add to the existing evidence supporting the use of electronic versions of the EORTC QLQ-C30 and associated EORTC modules to collect data in clinical trials when migrated according to best practice guidelines.

## Abbreviations

ECOG: Eastern Cooperative Oncology Group
EORTC QLQ-C30: European Organisation for Research and Treatment of Cancer Quality of Life-Core 30 item
EORTC QLQ-H&N35: EORTC Quality of Life Head and Neck 35 item
ePROs: electronic patient reported outcomes
G1: Group 1
G2: Group 2
HRQoL: health-related quality of life
PROs: patient reported outcomes
